# A Stereolithography-Based Modified Spin-Casting Method for Faster Laboratory-Scale Production of Dexamethasone-Containing Dissolving Microneedle Arrays

**DOI:** 10.3390/pharmaceutics16081005

**Published:** 2024-07-29

**Authors:** Martin Cseh, Gábor Katona, Szilvia Berkó, Mária Budai-Szűcs, Ildikó Csóka

**Affiliations:** 1 Institute of Pharmaceutical Technology and Regulatory Affairs, University of Szeged, Eötvös Str. 6, H-6720 Szeged, Hungary; cseh.martin@szte.hu (M.C.); berko.szilvia@szte.hu (S.B.); budai-szucs.maria@szte.hu (M.B.-S.); csoka.ildiko@szte.hu (I.C.); 2 3D Center, Center of Excellence for Interdisciplinary Research, Development and Innovation, University of Szeged, Tisza Lajos Blvd. 107, H-6725 Szeged, Hungary

**Keywords:** microneedle arrays, dexamethasone sodium phosphate, cutaneous drug delivery, ex vivo skin penetration, Raman mapping

## Abstract

Microneedle arrays (MNAs) consist of a few dozens of submillimeter needles, which tend to penetrate through the stratum corneum layer of the skin and deliver hardly penetrating drugs to the systemic circulation. The application of this smart dosage form shows several advantages, such as simple use and negligible pain caused by needle punctures compared to conventional subcutaneous injections. Dissolving MNAs (DMNAs) represent a promising form of cutaneous drug delivery due to their high drug content, biocompatibility, and ease of use. Although different technologies are suitable to produce microneedle arrays (e.g., micromilling, chemical etching, laser ablation etc.), many of these are expensive or hardly accessible. Following the exponential growth of the 3D-printing industry in the last decade, high-resolution desktop printers became accessible for researchers to easily and cost-effectively design and produce microstructures, including MNAs. In this work, a low force stereolithography (LFS) 3D-printer was used to develop the dimensionally correct MNA masters for the spin-casting method. The present study aimed to develop and characterize drug-loaded DMNAs using a two-level, full factorial design for three factors focusing on the optimization of DMNA production and adequate drug content. For the preparation of DMNAs, carboxymethylcellulose and trehalose were used in certain amounts as matrices for dexamethasone sodium phosphate (DEX). Investigation of the produced DexDMNAs included mechanical analysis via texture analyzer and optical microscopy, determination of drug content and distribution with HPLC and Raman microscopy, dissolution studies via HPLC, and ex vivo qualitative permeation studies by Raman mapping. It can be concluded that a DEX-containing, mechanically stable, biodegradable DexDMNA system was successfully developed in two dosage strengths, of which both efficiently delivered the drug to the lower layers (dermis) of human skin. Moreover, the ex vivo skin penetration results support that the application of DMNAs for cutaneous drug delivery can be more effective than that of a conventional dermal gel.

## 1. Introduction

Microneedle arrays (MNAs) have become a hot topic among researchers in the past decade as a novel approach in biomedical and healthcare applications [1,2,3]. These tiny structures can be utilized through alternative administration routes to obtain systemic efficacy in cases where conventional drug delivery is hardly or not accessible. However, MNA-based drug delivery systems have been widely studied through the transdermal administration route, and other applications, such as transmucosal, corneal, or buccal [4,5,6].

The greatest advantage of MNAs is the ability to penetrate the epidermis and cutaneously deliver drugs for both local therapy and central circulation. Furthermore, they can also enhance the effectiveness of topically administered drugs. Dissolving microneedles (DMNAs) could provide a novel treatment method for skin diseases such as atopic dermatitis and psoriasis by local delivery of different types of anti-inflammatory drugs, such as corticosteroids, to the skin under the stratum corneum [7,8].

By choosing the right needle morphology, the pain caused by these patches can be drastically decreased or totally ceased [9]. This property makes them attractive for patients with trypanophobia (fear of needles). In addition to the achievable higher patient adherence, MNAs can deliver poorly permeable hydrophilic drugs through the skin by physically penetrating the poorly permeable outer layer, the stratum corneum. The current literature describes five main types of microneedles: solid, coated, dissolving, hydrogel-forming, and hollow [2,10]. DMNAs volumetrically contain the drug formulation, which results in the highest amount of drug quantity per needle [10,11,12,13,14,15]. Another significant advantage is that potential breaking of the needles during the application does not cause complications—their general purpose is to dissolve in the upper dermis. Besides these, challenges also come with this type of MNA, as they have relatively weaker mechanical properties, more complex and time-consuming production, and hardly achievable drug content uniformity.

Various manufacturing strategies that involve multiple processing steps have been used to fabricate DMNAs [16]. The general steps of fabrication are the following: (1) preparation of DMNA master molds with 3D-printing, (2) casting of DMNA production molds, and (3) production of DMNAs loaded with drug. For the production of DMNAs, often water-soluble polymers are used to achieve successful micro-molding in the production mold and water-soluble drugs are required. When this was taken into account, dexamethasone sodium phosphate (DEX) was selected as the model drug to study the applicability of dermal DMNAs. DEX is a potent corticosteroid that is used topically to reduce inflammation and suppress immune responses in various skin conditions. Due to its hydrophilic nature, the penetration of the free form through the topical administration route is sufficient. DMNAs can enhance the delivery of DEX locally to affected areas of the skin in effective amounts without the need for substitutional per oral administration [17,18].

The present work focuses on the characterization of a modified spin-casting method to produce DEX-containing DMNAs based on carboxymethylcellulose/trehalose. This new method enables a faster production of DMNAs with better mechanical properties than were produced by the methods described by the previous works of different authors [14,15,19,20,21].

## 2. Materials and Methods

### 2.1. Chemicals

High-Temp V2 resin (Formlabs, Somerville, MA, USA) was obtained from Dental Plus kft. (Sopron, Hungary). Sylgard 184 polydimethylsiloxane (PDMS) was purchased from Farnell (Leeds, UK). Isopropyl alcohol (IPA) was acquired from Molar Chemicals Ltd. (Halásztelek, Hungary). Carboxymethylcellulose sodium (CMC), low viscosity (44.0 mPas at 25 °C, 2% *w*/*w* in H_2_O) was obtained from EMD Millipore Corp. (Burlington, MA, USA). D-(+)-Trehalose dihydrate (TRE) as well as dexamethasone sodium phosphate (DEX) were obtained from Merck Life Sciences Ltd. (Budapest, Hungary).

### 2.2. 3D-Printing of MNA Masters for Mold Production

Initially, the 3D designs of the MNA masters were obtained using Shapr3D (Shapr3D, Budapest, Hungary) CAD software version 5.37. The designs were then imported to PreForm (Formlabs, Somerville, MA, USA) slicer software version 3.29.1, followed by the necessary steps of the preparation of 3D-printing (orientation, support generation, setting the layer height). The models were printed on a Formlabs Form 3 printer (Formlabs, Somerville, MA, USA). The finished prints were washed in IPA for 6 min; supports were removed in this stage, followed by post-curing in a UV chamber for 2 h at 80 °C. The bottom of the final parts was ground with rough polishing paper to achieve better adhesion in the mold production step.

### 2.3. PDMS Mold Production for the Spin-Casting Method

Four of the printed masters were adhered to a glass surface with a drop of cyanoacrylate superglue. A PLA FDM 3D-printed ring was also adhered to the glass surface around the four masters to achieve the final shape of the master mold. Subsequently, the A and B components of the Sylgard 184 PDMS were mixed in a 10:1 ratio in the required amount for 10 min at room temperature, followed by degassing in a vacuum chamber. The crystal-clear, degassed mixture was then poured onto the 3D-printed masters. The curing of the PDMS was performed in an oven at 50 °C overnight. Four of the same molds were produced to fill all four of the centrifuge arms (Hermle Z323K, Hermle AG, Gosheim, Germany).

### 2.4. Spin-Casting of DMNAs

**Figure 1:**
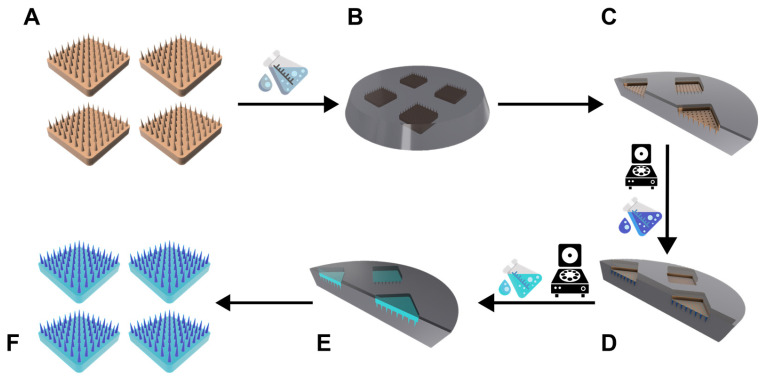
Graphical explanation of the spin-casting method: (**A**) 3D-printed masters, (**B**) PDMS elastomer poured on the masters during polymerization, (**C**) fully polymerized master mold with the DMNA negatives, (**D**) DEX spin-casted in the needle cavities of the mold, (**E**) spin-casted indifferent base gel for the baseplate, (**F**) final DMNA samples: DEX-containing needles (dark blue) with a DEX-less base plate (light blue).

As an initial step, the base gels were produced from CMC:TRE in a weight ratio of 70:30 [13]. A DEX-containing gel was made for the needle tips containing drugs in which the CMC:TRE mixture was used in 15% *w*/*w* and a DEX-less, indifferent CMC:TRE-only gel was made as the base of the DMNAs, which was 30% *w*/*w*. After mixing, the gels were degassed by centrifugation (Figure 1).

For the DMNA spin-casting method, the DEX-containing gel was poured into the molds in excess amounts and centrifuged (Hermle Z323K, Hermle AG, Gosheim, Germany) for 5 min at 20 °C at 3170 RCF to pull the gel into the needle cavities. Subsequently, the remaining excess gel was gently removed from the surface and a spin-drying process was obtained in the centrifuge for 15 or 45 min at 35 °C, 3170 RCF, according to the design of the experiment (DoE). The indifferent gel was then poured into the mold as the base of the DMNA and centrifuged for 2.5 or 3.5 h at 35 °C, 3170 RCF again, according to the design of the experiment. The finished DMNAs were gently removed from the molds, transferred to Eppendorf tubes, and stored in a desiccator until further use.

### 2.5. Design of Experiment

A two-level full factorial design for three factors was established to effectively investigate and filter the correlations between the independent variables (different production circumstances) and the properties of the product. The factorial design was developed with TIBCO Statistica^®^ 13.4 software (Statsoft Hungary, Budapest, Hungary) and was performed at three levels by manipulating the influencing parameters. The levels established on the basis of the experimental design are shown in Table 1.

### 2.6. Morphological Assessment of Needles

The morphology and dimensions of the samples were measured with a DSX510 optical 3D microscope (Olympus Corporation, Shinjuku, Tokyo, Japan) using a 10× optical magnification with fine acquisition settings. Top-view measurements and 3D reconstruction were made by the Olympus microscope based on the image stacking technique. Side-view images were also created using the same method.

### 2.7. Texture Analysis of DMNA Samples

An indirect mechanical stability test was performed on the basis of the work of Vora et al. [14], in which the height of 4 needles per sample was measured with the optical microscope. Subsequently, the needles were compressed with a force of 360 mN/needle parallel to the needle’s axes for 30 s with a TA XT Plus texture analyzer (Stable Micro Systems Ltd., Godalming, UK). Finally, the needle heights were measured again, followed by calculating the percentages of needle height reduction.

### 2.8. Qualitative Determination of Drug Content of DMNAs

Raman mapping was conducted to visually determine the relative content of the drug and its location within the samples [15]. Two measurement points were made within each sample: a single needle row was cut from the side part and a similar one from the middle section of the sample. The 1–1 needle was measured with its base to confirm the needle-selective drug localization. For the measurement, a ThermoFisher XRD Dispersive Raman microscope (Thermo Fisher Scientific Inc., Waltham, MA, USA) equipped with a CCD camera and a diode laser operating at a wavelength of 780 nm was used. A 1000 µm × 500 µm surface was analyzed with a step size of 50 µm. The exposure time was set at 4 s with an acquisition time of 4 s, for a total of 16 scans per spectrum in the spectral range of 3500 to 200 cm^−1^. Cosmic-ray and fluorescence corrections were performed. The Raman spectra were normalized to eliminate the intensity deviation between the measured areas. The OMNIC for Dispersive Raman 8.2 software (Thermo Fisher Scientific) was used for chemical evaluation. The individual spectrum of the unformulated DEX was used as a reference when profiling the chemical map.

### 2.9. Quantitative Determination of Drug Content of DMNAs

During qualitative studies of drug content, all needles (64) were removed from the indifferent base with a sharp blade from each sample and dissolved in 0.5–0.5 mL of purified water. Aliquots were measured with HPLC. The stationary phase was a Gemini NX C18 column (5 µm, 150 × 4.6 mm (Phenomenex, Torrance, CA, USA). The injection volume was 10 µL. The temperature was set at 40 °C. As mobile phases, a phosphate buffer (pH 6.8) (A) and acetonitrile (B) were used. For DEX determination, a 5 min isocratic elution in a 70:30 A-B ratio was applied. The eluent flow rate was 0.5 mL/min, and chromatogram detection was carried out at 254 ± 4 nm using a UV-Vis diode array detector. Data were evaluated using ChemStation B.04.03 software (Agilent Technologies, Santa Clara, CA, USA).

### 2.10. In Vitro Dissolution Studies

A special dissolution study method was applied to ensure that the dissolution medium contacted the needles only. The needles were pierced through a layer of Parafilm M. Subsequently, a pocket was formed by folding the parafilm back and hermetically closed by heat welding according to the method described by Larraeta et al. [18]. This pocket was fixed to a magnetic stirrer and the dissolution was conducted in 5 mL of 7.4 PBS with a 32 °C initial tempering at 100 rpm. Aliquots were withdrawn at 1, 3, 5, 10, 15, 30, and 60 min from the release medium and measured with HPLC.

### 2.11. Ex Vivo Permeability Studies

Permeation studies were conducted on ex vivo excised human skin acquired from a Caucasian female patient who underwent an abdominal plastic surgery procedure at the University of Szeged, Department of Dermatology and Allergology. Investigations were carried out with the approval of the Hungarian Medical Research Council (ETT-TUKEB, registration number: BMEÜ/2339-3/2022/EKU). A special 3D-printed applicator was developed and applied to the skin with a maximum force of 3270 ± 182 mN (*n* = 3). A constant force of 32 *n* was applied immediately for 30 s with the texture analyzer. Subsequently, the DMNA was left on the skin for 0.5 and 1 h. The treated skin samples were then frozen and divided into cross-sections (15 μm thick) with a Leica CM1950 cryostat (Leica Biosystems GmbH, Wetzlar, Germany). The cross-sections of the treated skins were measured with Raman mapping using the settings described above. The skin mapping was captured of an area of 150 × 1000 μm^2^, with a step size of 50 μm vertically and horizontally. The OMNIC for Dispersive Raman 8.2 software (Thermo Fisher Scientific) was used for chemical evaluation. As a reference, 200 mg hydrogels of 1% HPMC were used with the same amount of drug. The individual spectrum of the unformulated DEX was used as a reference when profiling the chemical map.

## 3. Results

### 3.1. Influence of Optimization Parameters on Height Reduction (Factorial Design)

The influence of selected optimization variables on the reduction in height of DMNAs was investigated using a factorial design response surface methodology. The individual main effects of the independent variables, X_1_—needle tip centrifugation time (min), X_2_—final centrifugation time of DMNA (h), and X_3_—nominal drug content (µg), on the reduction in needle height of DMNA (Y) were investigated (Table 2).

**Figure 2:**
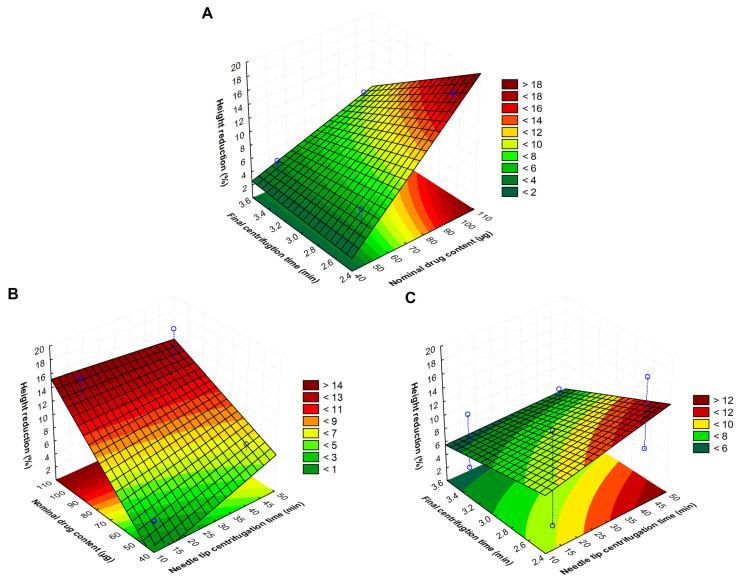
Fitted surfaces for the three independent variables that influence the needle height reduction value from different points of view: the effect of the centrifugation time (h) (X_2_) and the nominal drug content (µg) (X_3_) (**A**); the effect of the nominal drug content (µg) (X_3_) and the centrifugation time of the needle tip (min) (X_1_) (**B**); the effect of the centrifugation time (h) (X_2_) and the centrifugation time of the needle tip (min) (X_1_) (**C**) on the reduction in the needle height of DexDMNA (Y).

For easier interpretation of the significant impact of variables, the surface plot of independent variables versus needle height reduction may provide more information (Figure 2).

On the basis of the surface plots, it is clearly visible that reducing the nominal drug content, as well as the needle tip centrifugation time, the needle hight reduction can be reduced; however, decreasing the final centrifugation time increases the height reduction percentages. A linear equation can describe the individual main effects of the independent variables (X_1_, X_2_, and X_3_) on (Y), which was generated as follows:

Y = 8.37 + 0.99X_1_ − 2.13X_2_ + 4.37X_3_ − 0.64X_1_X_2_ − 1.11X_1_X_3_ − 1.72X_2_X_3_ (1)

The regression coefficient (R^2^) and the adjusted R^2^ of the surface plot were obtained as 0.99713 and 0.97992, respectively. Negative coefficients before the independent variables of the linear model indicate the favorable effect on Y. The significance of the effect of the investigated optimization variables (X_1_, X_2_, and X_3_) on Y is presented in Table 3.

Based on the ANOVA analysis, it can be concluded that the nominal drug content has a significant linear effect on height reduction. Increasing the DEX content, a more significant needle height reduction was observed, which indicates that increased drug content can reduce the consistency of the DMNA matrix.

### 3.2. Morphological Assessment of the 3D-Printed Masters and Final DMNA Products

**Figure 3:**
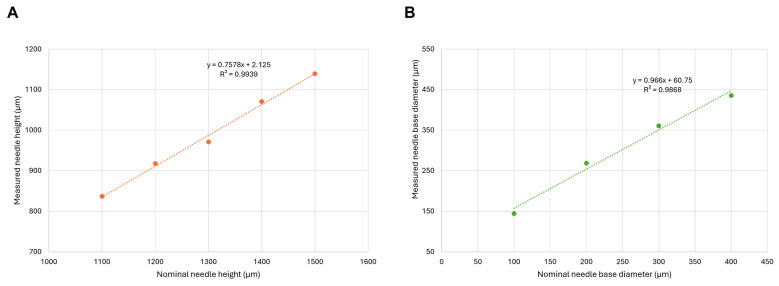
Correlation of nominal and measured printed needle heights (**A**) and base diameters (**B**).

Our criterion was to achieve conical needles with a needle height of 1000 µm and a base diameter of 400 µm. For this purpose, a calibration series with different needle heights (Figure 3A) and base diameters (Figure 3B) was prepared and the correlation between the nominal and measured morphological parameters was investigated. DMNAs were designed in a nominal needle height range of 1100 to 1500 µm with 100 µm steps, as well as the nominal base diameters being set in the range of 100 to 400 µm with 100 µm steps also. The measured dimensions were compared with the nominal values.

**Figure 4:**
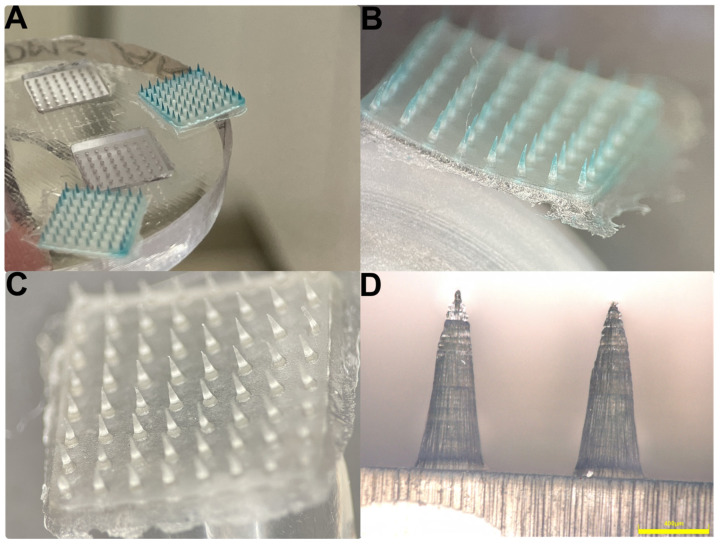
Optical microscopy images of the spin-casted DexDMNA samples: Preformulation samples for visualization of needle-selective drug content—needles stained by methylene blue (**A**); colored needles under stereomicroscope (**B**); actual DexDMNAs under stereomicroscope (**C**); side view of DexDMNA samples under the Olympus optical microscope (focus stacked) (**D**).

Based on the regression equation, the nominal values of needle hight (1317 µm) and base diameter (351 µm) were calculated that would result in the desired dimensions. To investigate the dimensional deviations between the printed masters and the spin-casted DMNAs, in addition to the previously mentioned parameters, the diameter of the apex and the volume of the needle were also determined for each DMNA. When comparing these parameters with the printed master, lower values were obtained in the case of spin-casted DMNAs; therefore, silicone molds were used to recast the MNAs from the original printer resin (Figure 4). These recast printer High Temp V2 resin-based MNAs were more similar in dimensions to the printed master than the DMNAs. The measured values for the different samples are shown in Table 4.

### 3.3. Texture Analysis of the DMNA Samples

**Figure 5:**
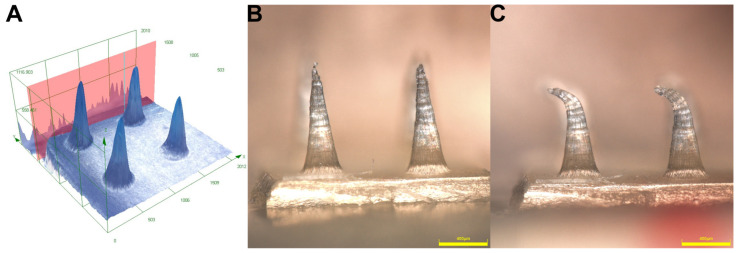
Three-dimensional reconstruction of the 4 needles measured in Olympus software version 3.1.7.1 (**A**); side microscopic view of DexDMNA samples before compression (**B**); and side microscopic view of DexDMNA samples after compression (**C**).

Texture analysis was performed with 360 mN/needle load to investigate the mechanical properties of DMNA (Figure 5). Texture analysis showed that all samples in which the needle drying time was 15 min were mechanically more stable compared to the samples with 45 min drying times (Table 5). The increased DEX content was also observed to significantly reduce the mechanical properties of the needles. The calculated statistics also support this state, which can be seen in the effect estimates table (Table 3) and the fitted surface graph (Figure 2). The same amount of force caused higher needle reductions in samples with higher DEX content, and it was also observed that the needles in the samples with increased DEX content broke very easily during the sample preparation process. Speaking of which, this was the cause of the failed measurements of DexDMNA_7—neither of the cutting attempts was successful in producing intact needles for the measurements. The samples made with the introduced modified spin-casting technique were compared to the methods already described by other authors using the same compositions. In order to compare the modified spin-casting method to those described in the literature, DexDMNA_1 and DexDMNA_5 were recast with a minor modification in the final drying process. The initial steps were the same as in the design of the experiment, but the final drying process, where the samples were spin-dried in the centrifuge, was modified to 15 min of room temperature, 3170 RCF, followed by 48 h of room temperature drying or 48 h of chamber drying at 35 °C. It was found that all the samples that were dried in RT or tempered to 35 °C for 48 h behaved worse mechanically than the equivalent samples made with the modified method.

### 3.4. Qualitative Determination of Drug Distribution of DMNAs

**Figure 6:**
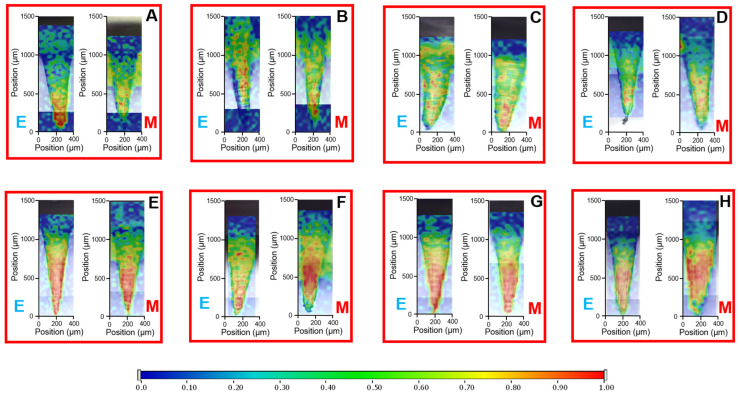
Results of Raman mapping of the drug content of different samples of DexDMNA: DexDMNA_1 (**A**), DexDMNA_2 (**B**), DexDMNA_3 (**C**), DexDMNA_4 (**D**), DexDMNA_5 (**E**), DexDMNA_6 (**F**), DexDMNA_7 (**G**), and DexDMNA_8 (**H**). The letters ‘E’ show the measurements of the edges of the samples, and ‘M’ show the middle region of the DMNAs.

High-resolution Raman mapping of the DMNAs was performed, selecting 1–1 needle from each, from two locations, the edge and the middle region of DMNAs, to investigate the homogeneity of drug distribution (Figure 6). Chemical maps showed that the drug was concentrated at the needle tips, supporting the efficiency of the spin-casting method. The red color in the chemical maps indicates the stronger existence of DEX, the green area shows the presence of DEX in lower concentration, whereas the blue color marks those regions of the map whose spectral resolution contains different spectra, characteristic of the DMNA base. Higher intensities in the DexDMNA_5–8 samples showed an increased drug content compared to DexDMNA_1–4. It was also observed that the drug concentration gradually decreased from the tip of the needle toward the base of the DMNA.

### 3.5. Quantitative Determination of Drug Content

HPLC analysis of the DMNAs revealed that some fraction of the drug was also localized in the base. DMNAs with 50 µg nominal DEX content of 50 g (DexDMNA_1–4) indicated ~39–60% of drug content in the needle tips; however, DMNAs with 100 µg of nominal drug content showed a more concentrated (~64–79%) drug distribution in the needle tips (Table 6). These findings also support the increased drug content and reduced the consistency of DMNA; therefore, in the case of DexDMNA_5–8, a higher amount of drug could concentrate in the cavity of the master during spin-casting. Thus, the higher drug content DMNAs are more suitable for therapeutic applications.

### 3.6. In Vitro Drug Release Studies

**Figure 7:**
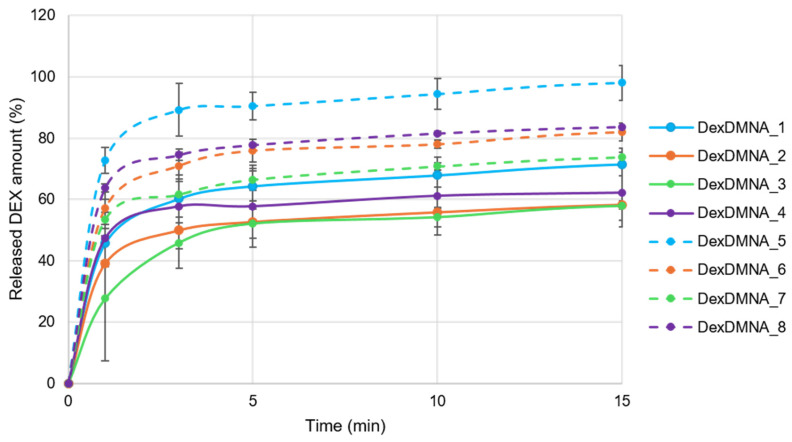
Drug release profiles of DexDMNAs.

In the drug release studies, the needles of DMNAs were pierced through a layer of Parafilm representing the human skin; therefore, the drug release medium was in contact only with the needle tips. The release study revealed burst release of the drug within the first three minutes, which can be attributed to the hydrophilic character of the DMNA matrix (CMC and TRE). In this short time period, 40–70% of the drug was released from DexDMNA_1–4 and more than 60% from DexDMNA_5–8. The dissolution rate was probably driven by the concentration gradient. The results are shown in Figure 7. The developed DMNAs represented fast dissolution properties, which can be advantageous for achieving rapid pharmacological effects through cutaneous administration.

### 3.7. Qualitative Ex Vivo Permeation Studies

**Figure 8:**
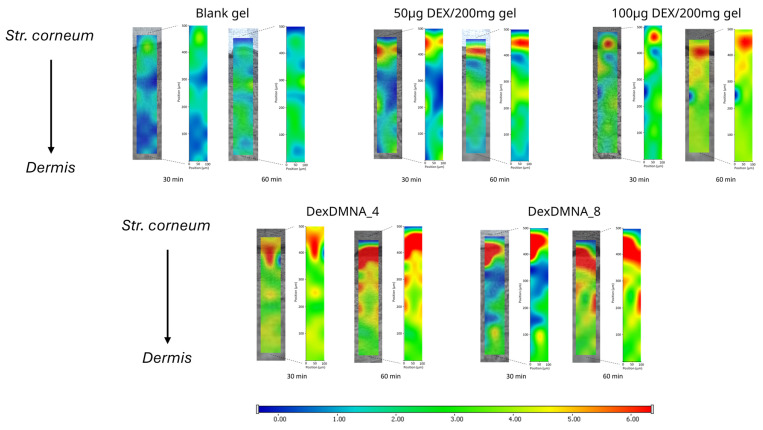
Ex vivo skin penetration studies of DexDMNAs.

Ex vivo permeation studies on human skin were performed to investigate the applicability of DMNAs as an innovative cutaneous drug delivery system (Figure 8). Raman mapping of skin specimens after 30 and 60 min of treatment revealed that DEX diffusion from reference gels (50 µg DEX/200 mg gel and 100 µg DEX/200 mg gel) was severely hindered by the stratum corneum, while the chemical maps showed a high penetration of drug in the lower layers of the skin (epidermis, dermis) treated with DMNA. DMNAs had high concentration peaks below the stratum corneum in the first 30 min, followed by a retained diffusion towards the dermis at 60 min. The reference gels also showed drug diffusion but with lower penetration intensities. Thus, the ex vivo results support that the application of DMNAs for cutaneous drug delivery can be more effective than a conventional dermal gel.

## 4. Discussion

Producing microneedle arrays is never a simple task, irrespective of whether we use any of the capable technologies due to their size and complexity. Although microneedling has been a hot topic in the past few years, there are no standard test methods or official regulations relating to them. This fact makes publishing and research even more difficult due to the different measurement methods.

As the morphological studies showed, a good MNA design and preformulation studies, along with CAD-to-print compensations, can result in the desired dimensions, or at least very close to them. It can be concluded that during the DMNA production, the water loss from the drying process causes an isotropic decrease in the sample dimensions (this was proved by recasting the samples from the printer resin from the same mold, where the final dimensions were close to the printed master’s dimensions), which should also be considered when designing the final geometry. However, this does not affect the drug loading efficiency because of the initial dimensions of the mold, which are exact negative copies of the printed master MNAs.

From the texture analysis of the DMNAs, it was concluded that the initial needle drying times and the DEX content affect the mechanical strength the most. However, the results of the DoE statistical analysis showed significance in the case of the DEX content (*p* < 0.05). The calculated coefficients indicate that modifying the first two factors (the needle and the final centrifugation times) may have a limited impact on the mechanical stability of the needles. Conversely, the composition itself shows significant potential for optimizing the product. It can be concluded that varying the centrifugation times to a relatively wide range did not result in a significant improvement or deterioration in the mechanical behavior to the extent that further research in this area would be warranted. This condition indicates that further investigations should prioritize factors related to the composition (polymer: sugar ratio, matrix gel concentration, drug content, etc.) over time-based centrifugation circumstances. It should be noted that this observation does not contradict the assertion that this spin-casting method produces samples with superior mechanical stability over conventional techniques. However, the increase in needle brittleness caused by DEX may be attributed to the plasticizing effect of DEX itself, which has yet to be proven in future work. The modified spin-casting method introduced in this work resulted in better performance DexDMNAs compared to the products made by conventional methods described previously according to texture analysis of the samples. Although the causal basis of this phenomenon has not yet been described, it is hypothesized that the constant centrifugal force during drying may change the structure of the sample—an investigation will be conducted in the future.

Localized needle-selective drug content was observed by high-resolution Raman mapping of the DexDMNAs, which is favorable for precise drug administration. The visualized drug content also helps to prove that the desired amount of drug is in the needles only, supported by a fully indifferent, drugless, cellulose-based platform. Qualitative measurements also highlighted the drawbacks of the needle-cutting sample preparation method; although, theoretically, this offers the most selective solution to determine the drug content in the needles, the brittleness and the size of the submillimeter needles cause them to bounce away. When these amounts are considered, even a single missing needle can cause high losses and deviations. By dissolving the full DexDMNAs, the measured content was near the desired level, with much lower deviations. However, needle separation methods would give the most precise results; simply cutting them without any bounce protection can result in false values—which happened in the initial measurements in this work.

Dissolution studies suggest that drug release is concentration-driven. Formulations with identical factors A and B behaved as pairs during dissolution studies, where the increased drug content resulted in higher dissolution rates, while the same sequence was retained within the two drug content groups.

Ex vivo studies prove the superiority of the DexDMNA system over the same drug-containing hydrogels, which supports the relevance of this administration in the future of medicine. Faster and more efficient delivery of drugs to lower skin levels can improve the efficacy of treatment and can contribute to a higher recovery rate of patients suffering from inflammatory dermal diseases compared to conventional dermal and transdermal formulations.

## 5. Conclusions

In this work a modified spin-casting method was introduced to produce DEX-loaded DMNAs that behave similarly mechanically, or better, compared to the other spin-casting methods. The study highlighted that this spin-casting method not only resulted in mechanically more stable DexDMNAs, but also reduced production times to 4–5 h, which is also a crucial aspect of personalized medicine.

## Figures and Tables

**Table 1:** Levels and variables of factorial design.

Independent Variables	Code	Levels	
		−1	+1
Needle tip centrifugation time (min)	X_1_	15	45
Final DMNA centrifugation time (h)	X_2_	2.5	3.5
Nominal drug content (µg)	X_3_	50	100

**Table 2:** The values considered of the influence parameters for 8 different preparations used to perform the factorial design.

Sample No.	Sample Name	Needle Tip Centrifugation Time (min)	Final DMNA Centrifugation Time (h)	Nominal Drug Content (µg)	Height Reduction (%)
1.	DexDMNA_1	15	2.5	50	1.97
2.	DexDMNA_2	45	2.5	50	6.84
3.	DexDMNA_3	15	3.5	50	1.84
4.	DexDMNA_4	45	3.5	50	5.33
5.	DexDMNA_5	15	2.5	100	15.79
6.	DexDMNA_6	45	2.5	100	17.4
7.	DexDMNA_7	15	3.5	100	9.93
8.	DexDMNA_8	45	3.5	100	7.84

**Table 3:** Effect of the optimization parameters on the height reduction of DexDMNAs produced by the spin-casting method. Statistical analysis: One-way ANOVA, α = 0.05. (* *p* < 0.05 significant, while n.s. means a non-significant effect).

Variable	Code	Significance	Effect of Variable on Height Reduction
Needle tip centrifugation time (min)	X_1_	n.s.	+
Final DMNA centrifugation time (h)	X_2_	n.s.	−
Nominal drug content (µg)	X_3_	* *p* < 0.05	+

**Table 4:** Measured morphological parameters of DexDMNAs.

	Diameter [µm] Mean ± SD (*n* = 2)	Height [µm] Mean ± SD (*n* = 2)	Apex Diameter [µm] Mean ± SD (*n* = 2)	Calculated Sum Needle Volume/Sample [mm^3^]
Printed master	410.5 ± 9.2	948 ± 43.8	31 ± 7.1	2.60
DexDMNA_1	391 ± 8.5	878.5 ± 30.4	20.5 ± 10.6	1.57
DexDMNA_5	396 ± 18.4	908 ± 38.2	33.5 ± 3.5	1.60
Spin-casted High Temp V2 resin	421.5 ± 3.5	992 ± 8.5	12 ± 5.7	2.76

**Table 5:** Height reduction values of the DexDMNAs prepared by the modified and previously described spin-casting methods.

Factor No.	Sample Name	Height Reduction Averages after 360 mN/Needle (Means ± SD; *n* = 12)) [µm]	Height Reduction Averages after 360 mN/Needle [%] (Means ± SD; *n* = 12)
1.	DexDMNA_1	16.83 ± 12.74	1.97 ± 1.51
2.	DexDMNA_2	45.83 ± 25.14	5.01 ± 0.73
3.	DexDMNA_3	15.88 ± 10.08	1.84 ± 1.12
4.	DexDMNA_4	48.36 ± 3.44	5.33 ± 0.28
5.	DexDMNA_5	139.25 ± 70.97 *	15.79 ± 7.95
6.	DexDMNA_6	164.17 ± 2.96 *	17.40 ± 0.26
7.	DexDMNA_7	Failed	Failed
8.	DexDMNA_8 *	71.67 ± 11.00 *	7.84 ± 1.23
9.	DexDMNA_RT_50	99.17 ± 37.70 *	9.26 ± 4.00
10.	DexDMNA_35C_50	184.33 ± 98.08 *	19.62 ± 10.18
11.	DexDMNA_RT_100	80.92 ± 23.00 *	8.98 ± 2.83
12.	DexDMNA_35C_100	196.28 ± 124.53 *	21.62 ± 12.97

* Significant differences are indicated with asterisks (* *p* < 0.05).

**Table 6:** Drug content of the different DMNAs with two different methods: needles cut from the base only dissolved and full DMNA dissolved.

Factor No.	Sample Name	Actual Drug Content (Needles Cut) [µg] (Means ± SD; *n* = 3)	Actual Drug Content (Full DMNA Dissolved) [µg] (Means ± SD; *n* = 3)
1.	DexDMNA_1	25.34 ± 4.66	46.14 ± 1.00
2.	DexDMNA_2	29.19 ± 6.72	47.78 ± 1.00
3.	DexDMNA_3	30.45 ± 2.74	48.11 ± 0.83
4.	DexDMNA_4	19.47 ± 0.93	47.71 ± 2.40
5.	DexDMNA_5	79.06 ± 3.12	104.51 ± 1.38
6.	DexDMNA_6	77.08 ± 8.90	103.91 ± 1.43
7.	DexDMNA_7	64.53 ± 5.98	100.79 ± 2.01
8.	DexDMNA_8	70.67 ± 7.47	105.38 ± 4.84

## Data Availability

The raw data supporting the conclusions of this article will be made available by the authors on request.
